# First evidence of SGPL1 expression in the cell membrane silencing the extracellular S1P siren in mammary epithelial cells

**DOI:** 10.1371/journal.pone.0196854

**Published:** 2018-05-02

**Authors:** Nadja Engel, Anna Adamus, Marcus Frank, Karin Kraft, Juliane Kühn, Petra Müller, Barbara Nebe, Annika Kasten, Guido Seitz

**Affiliations:** 1 Department of Pediatric Surgery, University Hospital Marburg, Baldingerstraße, Marburg, Germany; 2 Department of Cell Biology, University Medicine Rostock, Schillingallee, Rostock, Germany; 3 Department of Oral and Maxillofacial Surgery, Facial Plastic Surgery, Rostock University Medical Center, Schillingallee, Rostock, Germany; 4 Medical Biology and Electron Microscopy Centre, University Medicine Rostock, Strempelstraße, Rostock, Germany; 5 Complementary Medicine, Center of Internal Medicine, University Medicine Rostock, Ernst-Heydemann-Straße, Rostock, Germany; 6 Institute for Immunology, Friedrich-Loeffler-Institut, Federal Research Institute for Animal Health, Südufer, Greifswald-Insel Riems, Germany; Faculty of Medicine & Health Science, UNITED ARAB EMIRATES

## Abstract

The bioactive lipid sphingosine-1-phosphate (S1P) is a main regulator of cell survival, proliferation, motility, and platelet aggregation, and it is essential for angiogenesis and lymphocyte trafficking. In that S1P acts as a second messenger intra- and extracellularly, it might promote cancer progression. The main cause is found in the high S1P concentration in the blood, which encourage cancer cells to migrate through the endothelial barrier into the blood vessels. The irreversible degradation of S1P is solely caused by the sphingosine-1-phosphate lyase (SGPL1). SGPL1 overexpression reduces cancer cell migration and therefore silences the endogenous S1P siren, which promotes cancer cell attraction—the main reason for metastasis. Since our previous metabolomics studies revealed an increased SGPL1 activity in association with successful breast cancer cell treatment *in vitro*, we further investigated expression and localization of SGPL1. Expression analyses confirmed a very low SGPL1 expression in all breast cancer samples, regardless of their subtype. Additionally, we were able to prove a novel SGPL expression in the cytoplasm membrane of non-tumorigenic breast cells by fusing three independent methods. The general SGPL1 downregulation and the loss of the plasma membrane expression resulted in S1P dependent stimulation of migration in the breast cancer cell lines MCF-7 and BT-20. Not only S1P stimulated migration could be repressed by overexpressing the natural SGPL1 variant not but also more general migratory activity was significantly reduced. Here, for the first time, we report on the SGPL1 plasma membrane location in human, non-malignant breast epithelial cell lines silencing the extracellular S1P siren *in vitro*, and thereby regulating pivotal cellular functions. Loss of this plasma membrane distribution as well as low SGPL1 expression levels could be a potential prognostic marker and a viable target for therapy. Therefore, the precise role of SGPL1 for cancer treatment should be evaluated.

## Introduction

The bioactive lipid and second messenger sphingosine-1-phosphate (S1P) has emerged as key regulator in cancer progression by modulating a variety of cellular processes, such as proliferation, migration, platelet aggregation, or angiogenesis [1, 2]. Most studies have focused on S1P-synthesizing enzymes: the two S1P kinases (Sphk1, Sphk2), which are phosphorylating the pro-apoptotic lipid signaling molecule sphingosine. Several growth factors and steroid hormones, such as TGF-ß and 17ß-estradiol could be related to up-regulation of Sphk1 in cancer cells, producing high amounts of SP, which can bind extracellularly to five specific G-protein-coupled receptors, located on the cell surface (named S1P1, 2, 3, 4, 5) or intracellularly stimulate cell survival [3, 4]. Inhibiting the Sphk1 activity leads to a decrease in cancer proliferation and metastasis in mouse models and increases the efficacy of chemotherapy and radiotherapy [2, 5]. Consequently, Sphk1 is announced as a potential prognostic marker and a viable target for therapy, including that of breast cancer [6]. Considerably less attention has been paid to the S1P-degrading enzyme: sphingosine-1-phosphate lyase (SGPL1; EC 4.1.2.27) which irreversibly cleaves S1P into hexadecanal and ethanolamine phosphate and, is thus in a strategic position to regulate these processes by removing available S1P signaling pools, which means silencing the siren in order to prevent cancer cell migration to the blood vessels [7, 8]. Current research has demonstrated that ectopic expression of SGPL1 results in increased sensitivity to stress, including serum starvation [9], to platinum-based drugs, daunorubicin and etoposide [10], and irradiation [11] suggesting that SGPL1 may be a useful target for cancer therapy drugs.

SGPL1 belongs to the PLP (pyridoxal 5’-phosphate) -dependent carbon-carbon lyases and to the family of single-pass type III membrane proteins, and is located in the endoplasmic reticulum [12]. The luminal N-terminal domain is close to the transmembrane segment and the soluble PLP-binding domain. The larger, hydrophilic and catalytic domain faces the cytosol. Several mammalian tissues express SGPL1 in different activity states. While high activity appears in the small intestine, colon, thymus and spleen, moderate activity is found in liver, kidney, lung, stomach and testis. SGPL1 underlies a strict transcriptional and posttranslational regulation, enabling it to respond to environmental changes. Loss of SGPL1 enhances cell resistance to anticancer regimens and increases the ability of cells to acquire a transformed phenotype and become malignant. On the contrary, malignant cancer cells with moderate SGPL1 expression show sensitivity to cisplatin, daunorubicin and etoposide [10]. Inhibition of SGPL1 activity has profound effects on the immune system and lymphocyte trafficking. The sphingosine analog FTY720 is known to inhibit SGPL1 *in vitro* as well as *in vivo* [13]. However, SGPL1 promotes apoptosis through p53 and p38 tumor-suppressor signaling pathways, and therefore indicates that a SGPL1 downregulation in many cancer types is likely. Yet, an upregulation has been observed in some malignant tissues such as ovarian cancer [14]. Hence, the putative role as tumor-suppressor is not yet convincing. For this reason, we explored the SGPL1 expression, location and function in breast cancer cells and tissue in comparison with non-tumorigenic controls with the intent to identify the underlying regulative mechanisms. Thereby, we explored a novel SGPL1 expression in the cytoplasmic membrane of healthy breast cells which could prevent extracellular overstimulation of circulating S1P. Prevention of breast cancer as well as avoidance of breast cancer progression is of the utmost importance since the incidences are still the highest in women worldwide [15]. Effective treatment of this heterogeneous disease is dependent on histological subtype and receptor expression status. The majority (77%) of breast cancers are positive for estrogen, progesterone, and the human epidermal growth factor receptor-2 and therefore suitable for endocrine therapies with Tamoxifen, Anastrozole, or Trastuzumab [16, 17]. However, triple negative breast cancer (10–17%) lacking the expressions of these three receptors are difficult to treat, due to their multiple drug resistance. Therefore, our research on molecular levels was performed with two triple negative breast cancer cell lines (BT-20, MDA-MB-231) as well as one luminal receptor positive cell line (MCF-7). As non-tumorigenic, epithelial breast cells, MCF-10A as well as MCF-12A were chosen, presenting two immortal, non-transformed cell lines that share characteristics and features of basal progenitor cells [18].

## Results

### Breast cancer cells harbor low SGPL1 protein contents

**Fig 1:**
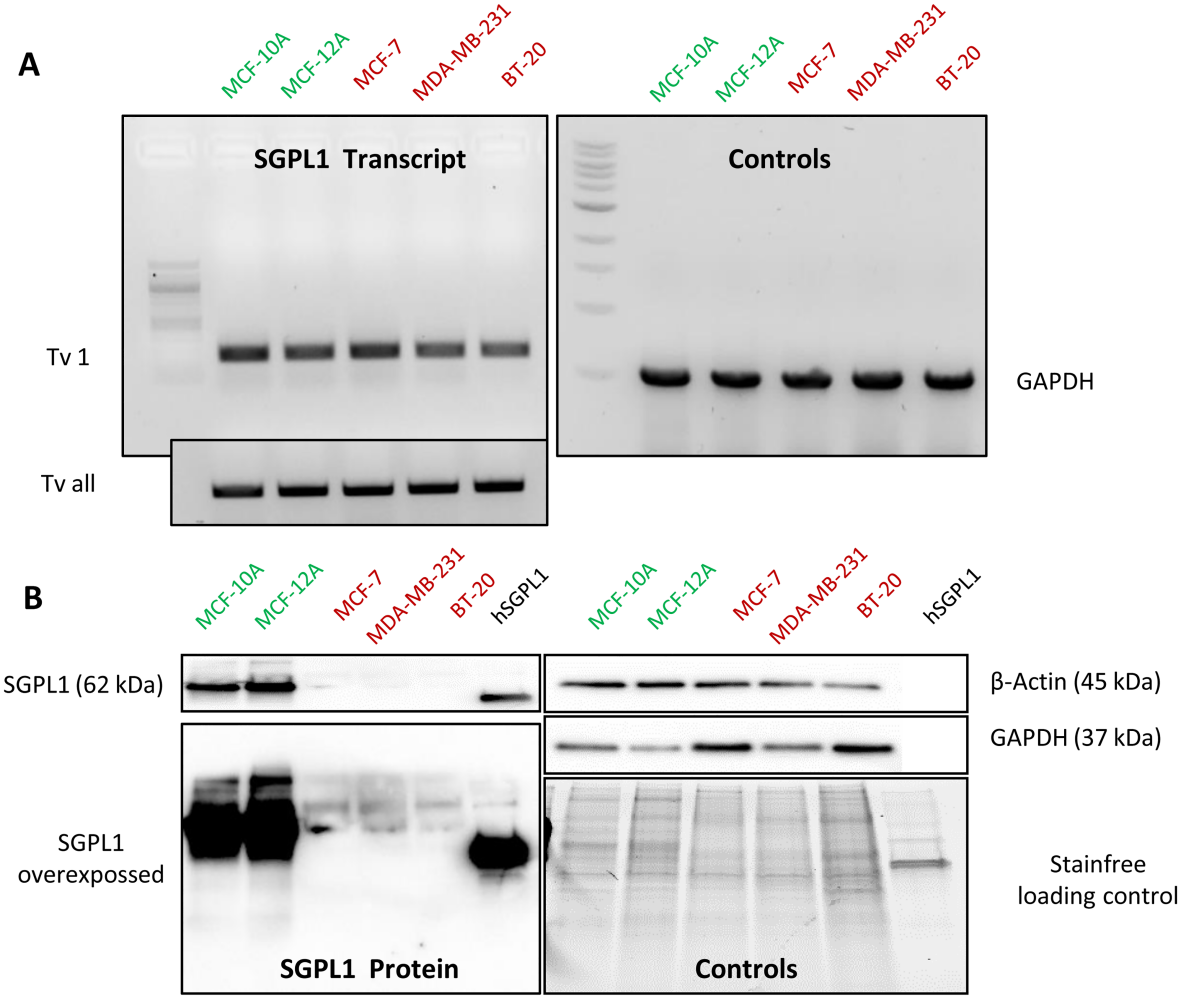
SGPL1 expression analysis. **A**: RT-PCR with SGPL1 specific primers revealed no significantly altered SGPL1 expression on transcript level for main coding SGPL1 transcript variant 1 (Tv1) as well for the sum of all NCBI annotated SGPL1 transcript variants (Tv all) in comparison with the GAPDH control. **B**: SGPL1 protein content in the 5 epithelial breast cell lines (20 μg soluble protein) parallel to 10 ng recombinant SGPL1 protein was visualized by western blotting. Non-malignant breast epithelial cell lines MCF-10A and MCF-12A showed strongly elevated SGPL1 protein expression levels. Loading controls were verified by labeling ß-actin and GAPDH proteins as well as by stain-free technology. Uncropped immune blots are shown in S1 Fig.

**Fig 2:**
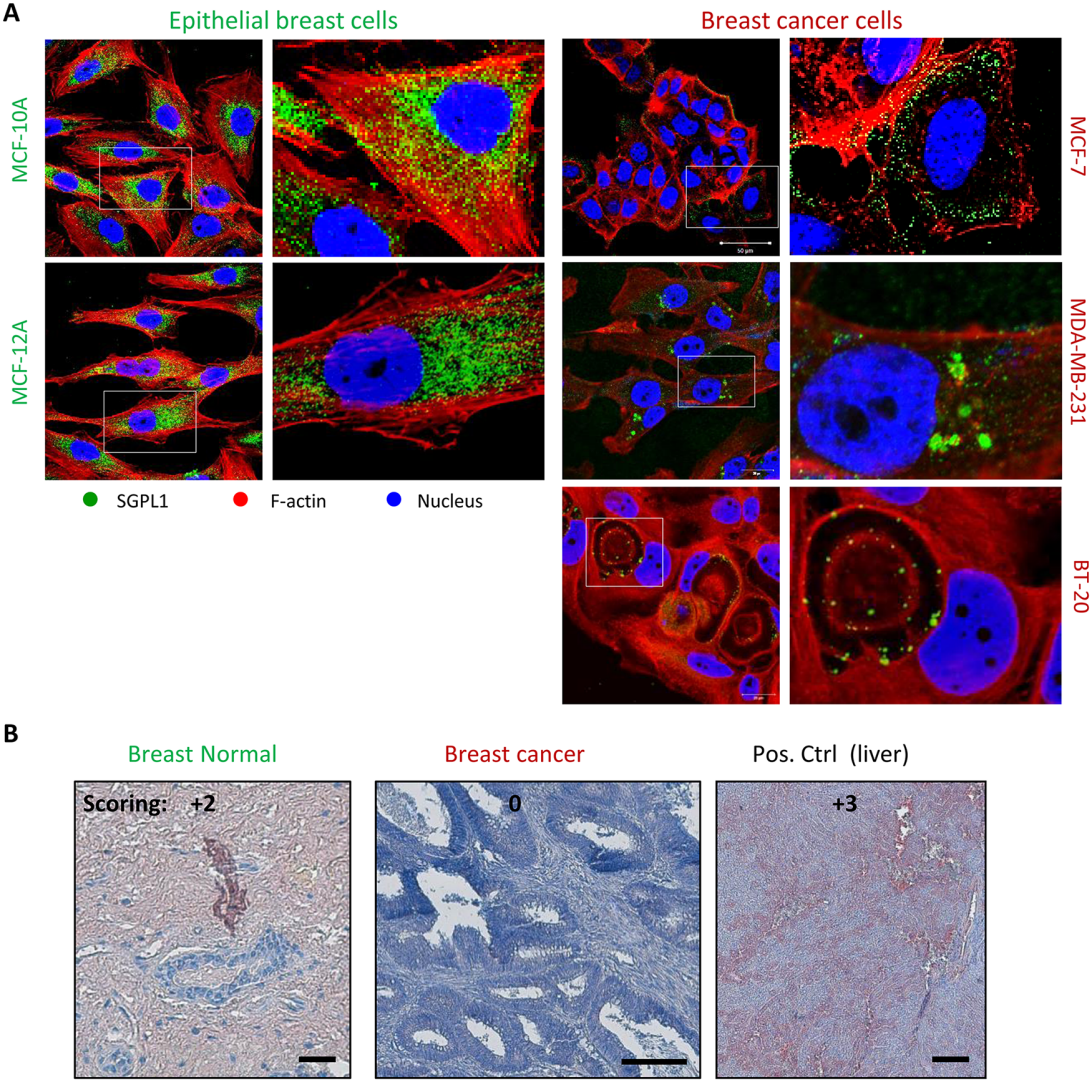
**A: Intracellular SGPL1 expression**. **A**: SGPL1 immunofluorescence staining (green) of two non-malignant breast epithelial cell lines (MCF-10A, MCF-12A) in comparison with three established breast cancer cell lines (MCF-7, MDA-MB-231, BT-20) co-localized with ß-Actin (red) and nuclei (blue). **B**: Representative images of SGPL1 immunohistochemistry staining in normal and breast cancer tissue samples in comparison with positive control organs (liver). SGPL1: reddish brown; Nuclei: blue. Statistical evaluation of *in vivo* SGPL1 protein expression was performed by semi quantitative scoring (0 = no, 1+ = low, 2+ = medium, 3+ = strong expression). Notably, breast cancer tissue and all cancer cell lines displayed SGPL1 downregulation compared to normal breast tissue and non-tumorigenic cell lines.

Two non-tumorigenic, epithelial breast cell lines MCF-12A and MCF-10A were chosen as control cell lines to compare the SGPL1 features with three conventional available breast cancer cell lines: MCF-7 (representing a luminal, hormone dependent subtype), MDA-MB-231 and BT-20 (representing a the most aggressive, triple-negative subtype with tendency for metastatic invasion). On transcript level, SGPL1 expression was not altered (Fig 1A). Primers, specific for the SGPL1 main coding transcript variant 1 (Tv 1, NCBI Reference Sequence: NM_003901.3) only, and primers for all coding transcript variants (Tv all) were designed for and used in RT-PCR as well as in qPCR. Nor significant differences occurred on transcript level. In contrast, SGPL1 protein content was significantly lowered in all three breast cancer cell lines, verified by western blotting (Fig 1B). As internal loading controls ß-actin, GAPDH and stainfree imaging of total soluble proteins were used. Furthermore, 10 ng of the recombinant expressed, and affinity chromatography purified human SGPL1 protein was loaded additionally on the immune blot to display the specificity of the SGPL1 antibody used in this study. Uncropped immune blots are shown in S1 Fig. Decreased SGPL1 expression in breast cancer cell lines was verified by immunofluorescence staining (Fig 2A). In addition, confocal imaging of SGPL1 localization revealed the SGPL1 protein in surrounding of the cell nuclei in the non-tumorigenic breast cells, indicating for an association with the endoplasmic reticulum (see S4 Fig). In MCF-7 breast cancer cells a much weaker expression with a more diffuse, cytosolic distribution of the SGPL1 protein was observed. Both invasive breast cancer cells displayed only small spots of aggregated SGPL1 proteins. Surprisingly, in BT-20 cells SGPL1 clusters could primarily be detected in the alveolar structures (oncosomes). The general lowered SGPL1 expression was verified in immunohistochemically stained human breast cancer tissues (Fig 2B). Moderate to high (+2 to +3) scores were detected in normal breast tissue samples whereas the majority of breast cancer samples were weakly stained or negative (Score 0 to +1). Additional immunohistochemical SGPL1 stainings can be found, for example, in the Protein Atlas database (www.proteinsatlas.org, S3A Fig). A few cases of breast cancers displayed moderate immunoreactivity. Normal breast tissue displayed moderate to high SGPL1 contents comparable to liver sections used as positive controls. However, a correlation between overall and relapse-free patients’ survival and the SGPL1 expression value was determined significantly by using R2: Genomics Analysis and Visualization Platform (https://hgserver1.amc.nl/cgi-bin/r2/main.cgi) (S3B Fig). Thus, low SGPL1 expression levels are an indication of poor patient’ prognosis.

### SGPL1 is associated with the cytoplasmic membrane

**Fig 3:**
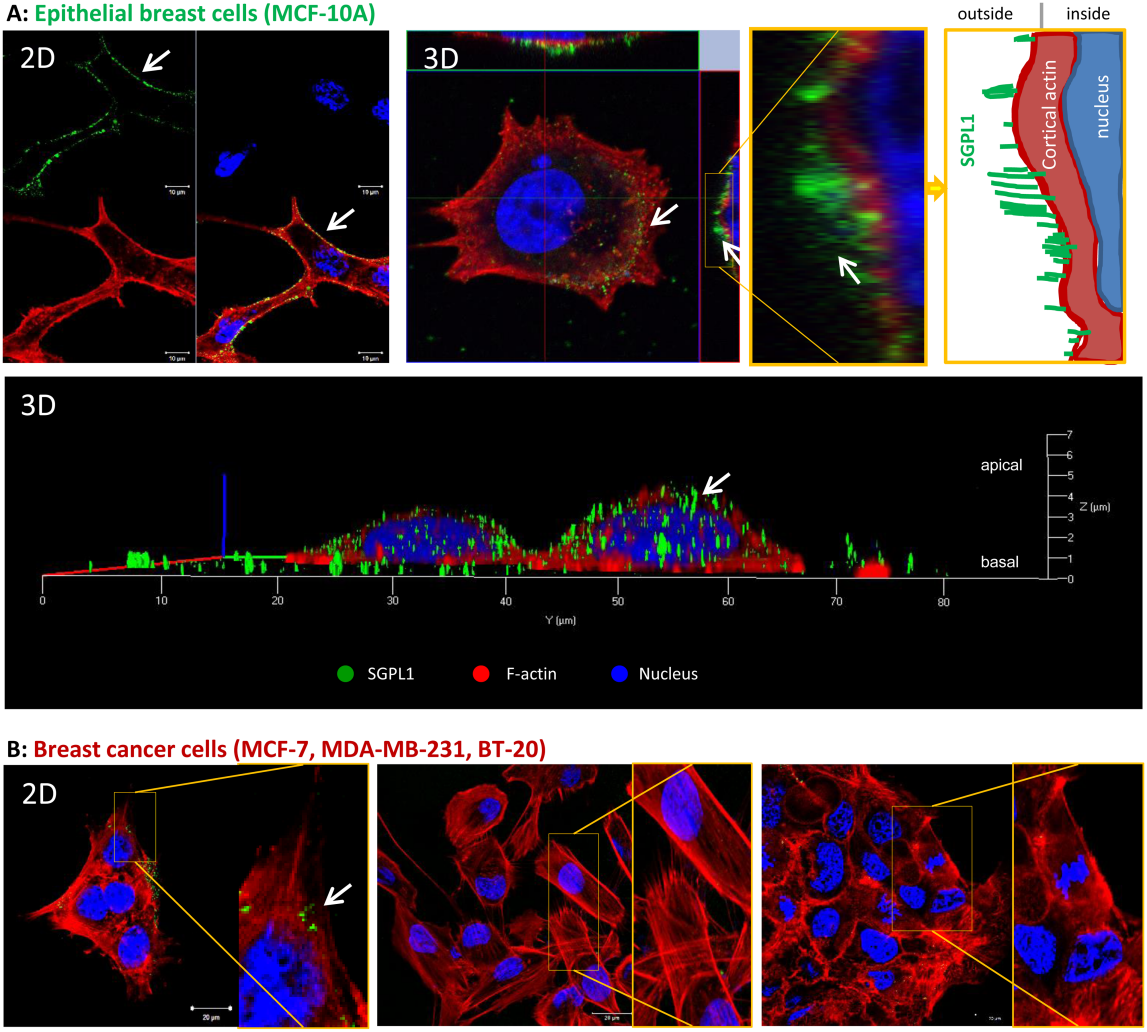
SGPL1 association with the plasma membrane. The living non-tumorigenic breast cell line MCF-10A (**A**) in comparison with the three breast cancer cell lines MCF-7, MDA-MB-231, and BT-20 (**B**) were labeled with a C-terminal anti-SGPL1 antibody without fixation and permeabilization, so that only cell surface and plasma membrane proteins can be labeled. After secondary labeling with Alexa488 dye, cells were fixed and counterstained with Phalloidin and Hoechst. SGPL1: green, F-actin: red, Nuclei: blue. **A**: Images of MCF-10A cells in 2D (upper panel) and 3D (lower panel) to visualize SGPL1 protein signals in direct association with the cell surrounding plasma membrane. 3D images were taken with Zeiss confocal laser scanning microscope. White arrows point to the green SGPL1 protein sitting on the cortical actin cytoskeleton. Additionally, a schematic view of SGPL1 in association with cell periphery is given. **B**: 2D images of SGPL1 expression in the breast cancer cell lines. Note that only non-tumorigenic breast cells show clear SGPL1 expression in cell periphery.

**Fig 4:**
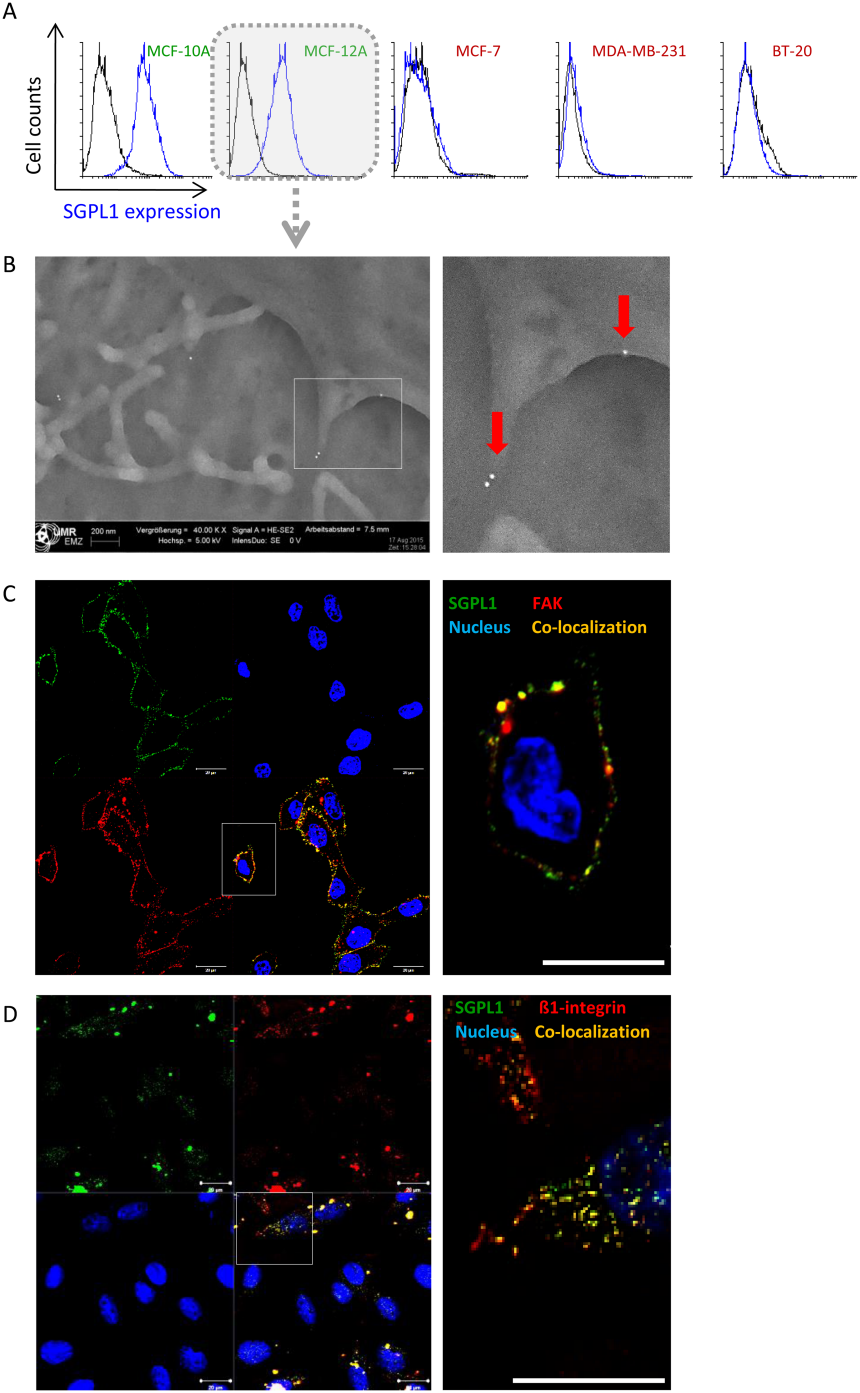
SGLP1 protein in association with the plasma membrane. A: SGPL1 labeling on living breast cells quantitatively measured by flow cytometry. Only the non-tumorigenic breast cells MCF-10A and MCF-12A exhibit SGPL1 signals on the cell surface. **B**: Scanning electron microscopy of gold-labeled SGPL1 proteins on the cell surface of the non-tumorigenic breast cell line MCF-12A. **C/D**: Co-localization studies of SGPL1 and known plasma membrane adhesion molecules for MCF-12A C: focal adhesion kinase (FAK) and D: ß1-integrin. SGPL1: green, FAK or ß1-integrin: red, membrane protein overlay: yellow, nuclei: blue. Bar = 20 μm.

The SGPL1 subcellular localization in the endoplasmic reticulum membrane was described previously [7, 8] and validated for epithelial breast cells in S4B Fig. By electronic annotations and proteomics approaches a SGPL1 localization in the plasma and mitochondrial membrane is likely (https://compartments.jensenlab.org; http://www.proteinatlas.org/ENSG00000166224-SGPL1/cell; http://www.genecards.org/cgi-bin/carddisp.pl?gene=SGPL1). Furthermore, the immunohistochemically stained non-malignant breast tissues revealed a SGPL1 association not only with the endoplasmic reticulum but also with the plasma membrane (Personal communication with pathologist Prof. Dr. Erbersdobler (Institute for Pathology, Rostock University Medical Center, Germany). To prove the prediction, live cell labeling with a C-terminal specific SGPL1 antibody was performed (Fig 3). Note that, staining’s presented in Fig 2 were performed after cell fixation and cell membrane permeabilization, while the staining’s in Fig 3 were done on living cells without fixation and permeabilization. As a result, the antibodies cannot enter the cell interior and thus, no intracellular proteins are labeled. Only cell surface and plasma membrane associated proteins can be marked by this method. 3D confocal imaging and remodeling of the non-tumorigenic breast cells revealed a green fluorescence signal on top of the actin fibers as it is known from adhesion molecules, for example the great group of integrins, which are anchored in the plasma membrane (Fig 3A). Further, a schematic view of the 3D cell cross sections is given on the right image of Fig 3A. The white arrows point to the green SGPL1 proteins in the cell periphery, which are sitting on the red labeled actin cytoskeleton. This green fluorescence signal at the cell surface was either lowered (MCF-7) or was absent in the triple negative breast cancer cells BT-20 and MDA-MB-231 (Fig 3B). This first evidence of a newly identified SGPL1 association with the plasma membrane was confirmed by additional experiments. By flow cytometry SGPL1 proteins were detected on the surface of the non-tumorigenic cell lines MCF-10A and MCF-12A (Fig 4A) but not in the breast cancer cells. Also scanning electron microscopy demonstrated a SGPL1 association with the cell surface in healthy breast cells (Fig 4B). Using gold-labeled secondary antibodies, small dots of SGPL1 proteins were detected at the edges of the filopodia in the non-tumorigenic MCF-12A cells, preferentially. No signals could be detected in breast cancer cell lines (S2 Fig). Finally, by co-localization studies with the membrane proteins focal adhesion kinase (FAK) and ß1-integrin the membrane integration of SGPL1 was hedged (Fig 4C and 4D). The green SGPL1 protein partially merged with the red labeled adhesion molecules, so that 50% fluorescence overlay could be detected. This observation proves that SGPL1 is a plasma membrane associated protein. A functional association with the adhesion protein cannot be assumed.

### S1P stimulation was prevented by overexpression of the natural SGPL1 variant

**Fig 5:**
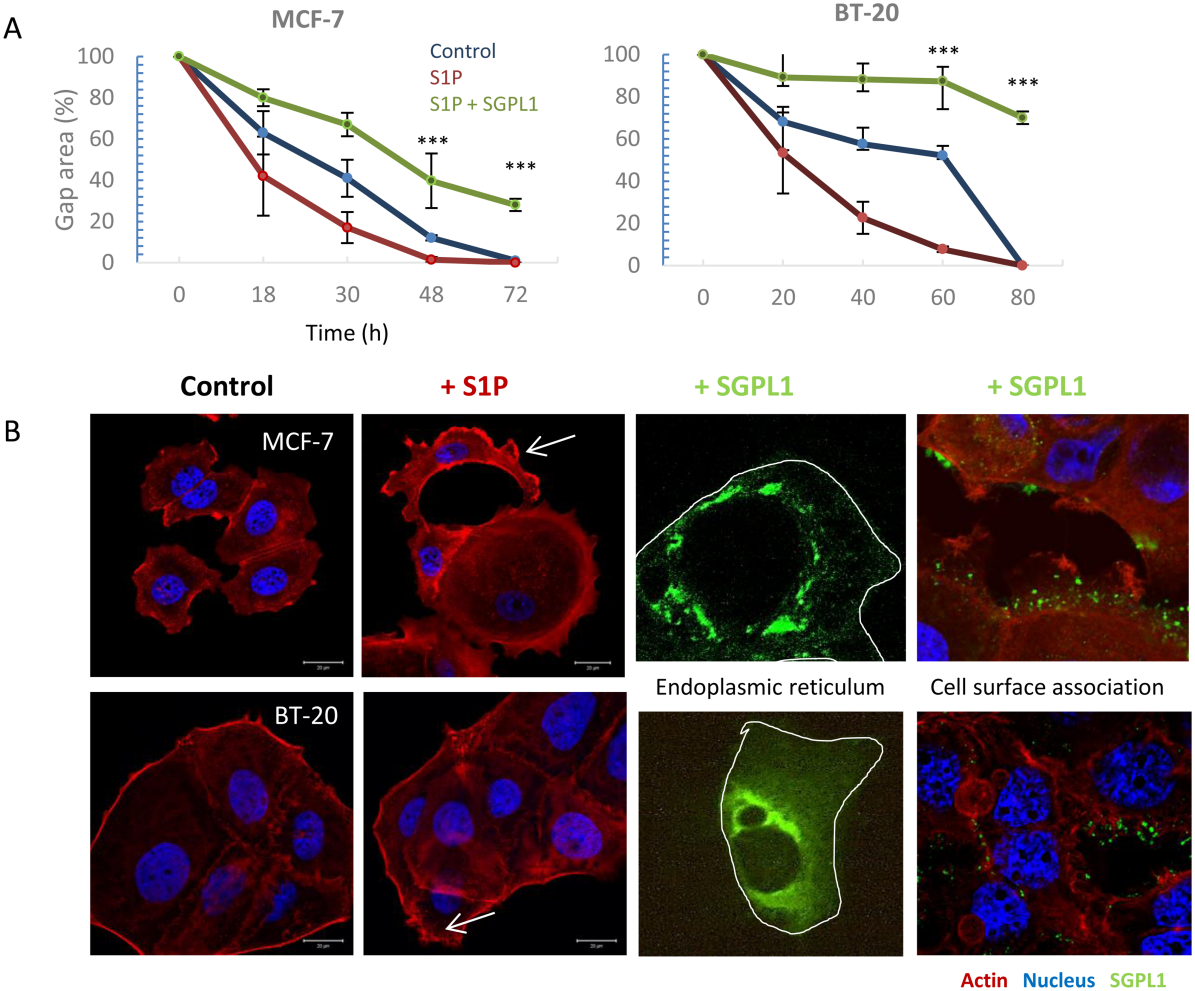
SGPL1 overexpression inhibits S1P induced migration. **A**: Enhanced migration speed after treatment with 1 μM sphingosine-1-phosphate (S1P) in the breast cancer cell lines MCF-7 and BT-20 (red line). SGPL1 overexpression inhibited the migration ability in the presence of S1P, significantly (green line). Mean ± SD, n = 5, ***P < 0.001, significantly different compared to vehicle treated cells, unpaired t-test. **B**: Morphological alterations of the cytoskeletal protein F-actin (red) after 24 h stimulation with 1 μM S1P. Breast cancer cells MCF-7 and BT-20 show an increased cortical F-actin formation and the formation of filopodia (white arrows). SGPL1 overexpression leads to SGPL1 enrichment within the cell in the surrounding of the nucleus and at the cell surface.

The results of previous studies suggest that the lack of SGPL1 membrane localization and overall lowered SGPL1 content permits uncontrolled extracellular S1P stimulation in the breast cancer cells which could lead to enhanced migratory activity. Though a classical migration assay, an increased migratory force by treatment with 1 μM S1P was verified for the cancer cell lines MCF-7 and BT-20 (Fig 5A). An influence on migration behavior of normal breast cells as well as the cell line MDA-MB-231 was not achieved. Additionally, alterations of the actin cytoskeleton were monitored under S1P treatment (Fig 5B). It was obvious that S1P strengthens the cortical actin and filopodia formation (white arrows). So far, we were able to conclude that low SGPL1 content and the missing SGPL1 expression in the outer membrane leads to migratory stimulation of the breast cancer cell lines MCF-7 and BT-20. By restoring the SGPL1 activity in these cancer cells, we checked if the S1P induced migration stimulation could be reverted (Fig 5A). Therefore, the SGPL1 cDNA of the healthy breast cells in frame with a constitutive promoter was stably transfected into MCF-7 and BT-20 cells (Fig 5B). Indeed, SGPL1 overexpression not only repressed the migration behavior to control levels but decreased the general motility of the cancer cells. Astonishingly, BT-20 cell migration ability was almost completely suppressed by SGPL1 overexpression. MCF-7 and BT-20 cells, which were transfected with a SGPL1-cDNA-GFP fusion plasmid revealed a strong co-localization with the endoplasmic reticulum and weak cytosolic and plasma membrane association (Fig 5B). Co-localization of SGPL1 with endoplasmic reticulum (ER) is presented in S4B Fig.

## Discussion

Under physiological conditions, high S1P concentrations (200 nM– 1 μM) circulate throughout in the bloodstream, and thus influence a variety of cellular processes: proliferation, migration, invasion, inflammation, and angiogenesis [19]. Tumor cells benefit from this circumstance, either by overexpressing the S1P synthesizing kinases (Sphk1, 2) or by repressing of the irreversible degrading lyase (SGPL1). Thereby, high S1P concentrations in the tumor environment are guaranteed and a continuous stimulation of the cancer cells is achieved. Several research groups have focused on the suppression of the Sphks (S1P generating kinases) activity or on the interception of the circulating S1P contents by the application of FTY720.

But high S1P concentrations are also needed to keep many cellular processes running, for example maturation of the vascular system, immune cell egress, stem cell survival, and cytokine production [20–24]. Systemic downregulation of S1P content in the human body leads to serious side effects. So, it is more likely that human cells control the continuous extracellular S1P stimulation by its degradation mediated by the SGPL1, which is localized in the endoplasmic reticulum. This consideration and the fact that our previously performed metabolome studies revealed an increased SGPL1 activity in association with successful cancer cell treatment *in vitro*, lead to more profound SGPL1 investigations in breast cancer cells [25, 4].

By western blotting and immunofluorescence staining (Fig 1) very low SGPL1 protein levels were found in all breast cancer samples, which correlates with other cancer studies. For example, SGPL1 is downregulated during intestinal tumorigenesis in the ApcMin/+ mouse model and in human colon cancer specimens compared to adjacent uninvolved tissues [10]. The loss of SGPL1 activity may contribute to tumorigenesis. This hypothesis is strengthened by the finding that SGPL1 is significantly downregulated in metastatic tumor tissues compared to primary tumors from the same patients [26]. Kaplan-Meier curves show a stringent correlation between low SGPL1 expression and poorer overall and relapse-free survival (S3B Fig)The low S1P contents could additionally be confirmed by immunofluorescence which also indicates an atypical expression particular in the two triple-negative breast cancer cell lines. MDA-MB-231 showed widely clustered SGPL1 proteins. BT-20 cells revealed an ectopic SGPL1 expression in the alveolar structures; the association with the endoplasmic reticulum was diminished. Since SGPL1 is a single-pass type III membrane protein, integration into other cellular membranes is conceivable. Free available databases (UniProtKB, Reactome) also affirmed the plasma membrane as potential subcellular localization. The co-localization of the SGPL1 protein with the plasma membrane was confirmed by immunofluorescence labeling of living epithelial breast cells (Fig 3). The highest SGPL1 expression in association with the plasma membrane was detected in the non-malignant breast cell lines MCF-10A and MCF-12A. Here, the SGPL1 proteins are sitting, like most of the adhesion molecules, on the F-actin fibers. Therefore, we decided to perform co-localization staining’s with both, the plasma membrane integrated protein ß1-integrin and the focal adhesion kinase (FAK) which connects the integrins with actin fibers. Indeed, co-localization of SGPL1 with adhesion molecules could be achieved (Fig 4). The newly identified SGPL1 plasma membrane localization was further verified by flow cytometry and scanning electron microscopy. The influence of the plasma membrane association on tumor physiology was estimated in two experiments: a) stimulation of the migration by the application of extracellular S1P and b) restoration of the SGPL1 function by a stable overexpression of the natural SGPL1 variant. The migration speed of the breast cancer cell could be increased during the incubation of 1 μM S1P. Overexpression of the native SGPL1 isoform not only neutralized the S1P effects, it also significantly inhibited the migration (Fig 5). These results proved the theory that a functional SGPL1 expression in plasma membrane combined with a high intracellular SGPL1 expression can silence the S1P siren, in the luminal breast cancer cell line MCF-7 and the triple-negative one, BT-20. To this end, we could demonstrate the first evidence of SGPL1 plasma membrane location in non-malignant breast epithelial cells, silencing the extracellular S1P siren *in vitro*, and thereby regulating pivotal cellular functions. Loss of this plasma membrane distribution as well as low SGPL1 expression levels could be a potential prognostic marker and a viable target for therapy. Therefore, the precise role of SGPL1 for cancer treatment should be further evaluated.

## Material and methods

### SGPL1 antibodies

Several SGPL1 (NCBI Accession: NM_003901, NP_003892.2) antibodies were verified for western blotting, immunofluorescence staining and flow cytometric analysis. Anti-SGPL1 specificity was enabled by specific binding to a recombinant expressed human SGPL1 protein. For western blotting experiments the rabbit anti-SGPL1 antibody (MaxPab^®^; ABIN948744 from www.antikoerper-online.de), raised against the full length SGPL1 amino acid sequence (AA 1–568), was chosen. For cytoplasmic membrane association, the polyclonal rabbit anti-SGPL1 antibody, recognizing a C-terminal epitope: amino acid 131–430 (cytoplasmic domain) sc-67368, Santa Cruz, USA was used.

### Cell culture conditions

All cell lines were purchased from ATCC (www.atcc.org) and maintained at 37 °C and in a 5% CO_2_ atmosphere. The non-tumorigenic mammary epithelial control cell lines MCF-10A (ATCC^®^ CRL-10317^™^) and MCF-12A (ATCC^®^ CRL-10782^™^) were grown in Dulbecco’s modified Eagle’s medium Ham’s F12 without phenol red (Invitrogen, Darmstadt, Germany) containing 10% horse serum (PAA Laboratories GmbH, Munich, Germany), and the Mammary Epithelial Cell Growth Medium Supplement Pack (Promo Cell, Heidelberg, Germany) including 0.004 ml/ml bovine pituitary extract, 10 ng/ml epidermal growth factor (recombinant human), 5 mg/ml insulin (recombinant human), 0.5 mg/ml hydrocortisone and 1% gentamycin (Ratiopharm GmbH, Ulm, Germany). The breast cancer cell lines MCF-7 (ATCC^®^ HTB-22^™^; estrogen and progesterone receptor positive), BT-20 (ATCC^®^ HTB-19^™^) and MDA-MB-231 (ATCC^®^ HTB-26^™^), both triple negative cancer cell lines were cultured in Dulbecco’s modified Eagle’s medium (Gibco, Paisly, UK) with 10% fetal bovine serum (PAN Biotech GmbH, Aidenbach, Germany) and 1% gentamicin (Ratiopharm GmbH, Ulm, Germany). Confluent cells were treated with 0.05% trypsin– 0.02% EDTA. The medium was changed every 2 days. Cell line authentication was performed by Seqlab Sequencing Laboratories (Göttingen, Germany).

### Transcript analysis

Total RNA from cultured cells was extracted with the NucleoSpin^®^ RNA II Kit (Machery-Nagel, Düren, Germany) and cDNA was produced from 2.5 μg of RNA with the First Strand cDNA Synthesis Kit (Thermo Fisher Scientific Inc., Rockford, IL, USA). qRT and RT-PCR was performed with the SGPL1 primer pair for transcript variant 1 (Tv1) fw: 5’-ATGCCTAGCACAGACCTTCT-3‘ and rv: 5’-CTTCCTGGTGAGCTTAAAACA-3’ amplifying a 240 bp fragment in the C-terminal region of the SGPL1 coding sequence. Primers flanking Tv1, Tv2 and all other NCBI annotated transcript variants were fw: 5’-ACTGCTCGCTTCCTCAAGTC-3’ and rv: 5’-GTGACAGTGTCGGTGCTGTA-3’ producing a 392 bp fragment. cDNA was mixed with iTaq^™^ SYBR^®^ Green Supermix (Bio-Rad Laboratories Inc., Hercules, USA) and analyzed on iQ^™^5 Multicolor Real-Time PCR Detection System (Bio-Rad, München, Germany). Relative gene expression was normalized by using two housekeeping genes: GAPDH fw: 5’-CAAGGTCATCCATGACAACTTTG-3’ and rv: 5’-GTCCACCACCCTGTTGCTGTAG-3’, and ß-actin fw: 5´-GGGCATGGGTCAGAAGGATT-3´ and rv: 5´-GAGGCGTACAGGGATAGCAC-3´.

### Protein analysis

Western blotting procedure was performed as described previously [27]. Recombinant protein of human sphingosine-1-phosphate lyase 1 (SGPL1) was produced with TrueORF clone, RC208705 encoding the full-length human SGPL1 with C-terminal DDK tag, from human HEK293 cells (GenBank accession: NM_003901, Predicted molecular weight: 63.3 kDa, purchased from OriGene Technologies, Inc.Rockville, USA; CAT#: TP308705. For protein detection, primary antibodies (MaxPab^®^; ABIN948744, USA; β-Actin #4970: Cell Signaling, USA; GAPDH: sc-137179, Santa Cruz, USA) were incubated overnight at 4°C followed by labeling with a horseradish peroxidase (HPR)-conjugated secondary antibody (Cell Signaling, USA) for 1 hour at room temperature. Protein signals were visualized by using SuperSignal West Femto Chemiluminescent Substrate (Pierce Biotechnology, Rockford, USA) for detection of peroxidase activity from HRP-conjugated antibodies. Band intensity was analyzed densitometrically with the Molecular Imager ChemiDoc XRS and Image Lab 3.0.1 software (Bio-Rad, USA). Protein detection was repeated at least three times with individually prepared cell lysates from independent passaged cells.

### Immunofluorescence and immunohistochemistry

The basic procedure for the immunofluorescence staining was described in Engel et al. 2012, 2014 [28, 29]. Cells grown on Menzel-Gläser coverslips (Thermo Fisher Scientific Inc., Schwerte, Germany) or in Ibidi dishes/ slides (Ibidi GmbH, Martinsried, Germany) with glass bottoms were fixed in 4% paraformaldehyde (Santa Cruz, Dallas, USA), permeabilized with 0.1% Triton X-100 (Santa Cruz, Dallas, USA) and labeled with anti-SGPL1 primary antibody (SGPL1: sc-67368, Santa Cruz, USA) and Alexa Fluor 488 dye secondary antibody (Thermo Fisher Scientific Inc., USA). Co-localization experiments were performed with Phalloidin-Alexa 596 (Invitrogen), focal adhesion kinase (FAK) primary antibody (#3285, cell signaling, USA), ß1-integrin (#9699, cell signaling, USA) or with cell-permanent ER-Tracker^™^ Green dye (BODIPY^®^ FL glibenclamide, Molecular Probes, USA) for live cell imaging. Finally, all samples were counter-stained with Hoechst (PanReacAppliChem, Darmstadt, Germany). Stainings were investigated with an inverted confocal laser-scanning microscope (LSM780, Carl Zeiss, Jena Germany). Notably, images were taken at identical device settings to guarantee comparable results. The image processing was carried out using ZEN 2011 (Carl Zeiss Jena GmbH, Jena, Germany). The expression intensity and distribution of SGPL1 protein was determined in healthy (GTX24324, GeneTex, Irvine, USA) and tumorigenic (GTX24701, GeneTex, Irvine, USA) human breast tissue. Murine liver served as control. Tissues were formalin-fixed, paraffin-embedded, cut, labeled with a SGPL1 primary antibody (SGPL1: sc-67368, Santa Cruz, USA) in a 2% BSA solution followed by a secondary antibody—labeled, polymer—horseradish peroxidase (HRP) (Dako Envision+ Kit; Dako, Glostrup, Denmark). AEC (3-amino-9-ethylcarbazol) served as chromogenic agent. All sections were counterstained with Mayer’s hemalum solution (Merck KGaA, Darmstadt, Germany). Each section was digitalized by using an AxioImager. M2 equipped with an integrated XY-scanning device and a MRc camera (all from Carl Zeiss Microscopy GmbH, Göttingen, Germany). Statistical evaluation of *in vivo* SGPL1 protein expression was performed by semi quantitative scoring (0 = no, 1+ = low, 2+ = medium, 3+ = strong and 4+ = very strong expression).

### Scanning electron microscopy

General scanning electron microscopy method was described previously in Engel et al., 2016 [28]. For the visualization of SGPL1 cell membrane association, cells were grown on coverslips to a confluency of 80%. After washing with PBS with Ca^2+^ and Mg^2+^, unspecific binding sites were blocked with 5% goat serum (PAN Biotech, Germany). Primary labeling with SGPL1 antibody (1:100 dilution of SGPL1 in 5% goat-PBS; sc-67368, Santa Cruz, USA) was performed at room temperature for 1 h. Secondary labeling with BBI gold anti-mouse antibody (1:200 dilution in 5% goat-PBS, 0.1% fish skin gelatin (BBI), 0,1% Tween (VWR, Germany); EM GMHL, 15 nm, BBI Solutions Cardiff, UK) was also performed for 1 h. After washing, cells were fixed with 2.5% glutaraldehyde in 0.05 M HEPES buffer. Samples were dehydrated in an ascending ethanol series and critical point dried using CO_2_ as an intermedium with the EMITECH 850 critical point dryer (Emitech Ltd. Ashford, UK). Carbon coating was done with the carbon coater SCD500 (Leica, Germany). Images were taken with a field emission scanning electron microscope (Zeiss Merlin VP Compact) using an acceleration voltage of 5 kV for imaging.

### Surface expression by flow cytometry

SGPL1 surface expression was measured by flow cytometry according to the measurements of other cell surface markers e.g. ß1-integrin, already described [29]. Briefly, after trypsinization, cells were washed with PBS (with 0.133 g/l CaCl_2_•2H_2_O and 0.1 g/l MgCl_2_•2H_2_O, Sigma, Germany) and then incubated with 100 μl of the rabbit anti-SGPL1 antibody, recognizing a C-terminal epitope: amino acid 131–430 (cytoplasmic domain) sc-67368, Santa Cruz, USA for 1 h at room temperature. Thereafter, cells were washed and secondarily labeled with Alexa Fluor 488 dye secondary antibody (Thermo Fisher Scientific Inc., USA) for 1 h at room temperature in the dark. After a final washing step with PBS, cells were diluted in 300 μl CellFix (Beckman Coulter, USA). As control the whole staining procedure was carried out without the primary antibody—PBS was used instead. 10,000 events were recorded for each measurement of SGPL1 surface expression and repeated three times. Results were calculated with the software FlowJo (https://www.flowjo.com).

### Migration analysis

Influence on migration was conducted on all five cell lines and stable SGPL1-transfected MCF-7 and BT-20 sorted cells, pre-incubated in assay medium for 48 h adaption in 6-well plates (Greiner, Germany). A defined gap was made by Ibidi culture inserts (μ-Dish 35 mm; Ibidi GmbH, Martinsried, Germany) following the instructor’s recommendations. When cell layers reached confluence, the culture insert was removed and cells were treated with 1 μM S1P or control (vehicle, MeOH) in assay medium (DMEM, 10% charcoal stripped fetal bovine serum (PAN BiotechGmbH, Germany). Medium was changed every day. Gap closure was analyzed as described previously [28].

### SGLP1 expression clones

A SGPL1-cDNA-GFP-tagged clone (NCBI Accession: NM_003901, NP_003892.2) was purchased from OriGene (#RG208705, Rockville, USA). Plasmid map is shown in Fig 4B: Sequencing of the full-length transcript variant was carried out with two vector sequencing primer by Seqlab Laboratories (Germany). 3x10^6^ cells of breast cancer cell lines MCF-7 and BT-20 were transfected with 1 μg/ml SGPL1-DNA plasmid using the CLB-Transfection System (Amaxa Nucleofection 6097129; Lonza, Cologne, Germany) according to the manufacturer´s instructions. As positive control the Pmax GFP^™^ vector (Lonza, Cologne, Germany) was used. After 24 h, transfection efficiency was controlled with a confocal laser-scanning microscope. To generate stable transfected cells, GFP negative cells were eliminated by fluorescence-based cell sorting (BD FACSAria, Heidelberg, Germany).

## Statistical analysis

All experiments were replicated at least three times with individually passaged cells, and data sets were expressed as means ± standard deviations (SD). Statistical significance was determined by the unpaired student’s t-test or one-way ANOVA (*** P < 0.001; ** P < 0.01; * P < 0.05) using the software Graphpad Prism Version 5 (http://www.graphpad.com/scientific-software/prism/) or Excel 2016.

## Supporting information

S1 Fig Uncropped SGPL1 immune blots und the stainfree loading control (A) of Fig 1B. B: Normal exposure time for detection relative to the saturation of the pixel. C: Overexposed SGPL1 immuno blot.
(TIF)

S2 Fig Verification of SGPL1 association with the plasma membrane of MCF-10A cells by flow cytometry. A: Gating of MCF-10A cells. B: Dilution series of primary (anti-SGPL1; 1:50, 1:100) and secondary (anti-rabbit Alexa488; 1:100, 1:200) antibodies to prove specific binding of the SGPL1 antibody. MCF-10A cells incubated with secondary antibody only, functioned as negative control (red histogramm). All signals with an FITC-A+ signal higher than log10^3^ were counted as postive events. (Data for the 1:50 prim./ 1:100 sec. Ab. dilution are shown in the blue histogramm; for 1:50 prim./ 1:200 sec. Ab. dilution in the orange histogramm; for 1:100 prim./1:100 sec. Ab. dilution in the green histogramm and 1:100 prim./ 1:200 sec. Ab. dilution in the dark green histogramm.) The 1:50 dilution of the primary (SGPL1) and 1:100 dilution of the secondary Alexa488-labeled antibody were considered as the effective ones and were used for the experiment.
(TIF)

S3 Fig **A**: SGPL1 expression status in healthy and cancer breast tissues, e.g. http://www.proteinatlas.org/ENSG00000166224-SGPL1/pathology. **B**: SGPL1 down-regulation is correlated with overall and relapse free survival of breast cancer patients. For example, you can check the online tool R2 for correlation analysis (https://hgserver1.amc.nl/cgi-bin/r2/main.cgi). The following Kaplan Curves demonstrate impressively that low SGPL1 expression leads to poorer overall and relapse-free survival.
(TIF)

S4 Fig **A**: Map of the SGPL1-ORF expression vector. **B**: Co-localization studies of SGPL1 with the endoplasmic reticulum. For further studies see http://www.proteinatlas.org/search/SGPL1. **C**: Scanning electron microscopy of gold-labeled SGPL1-proteins in the breast cancer cell line MCF-7 showed no signals.
(TIF)
