# Supplementary figures and images for: First evidence of SGPL1 expression in the cell membrane silencing the extracellular S1P siren in mammary epithelial cells
Source: PLoS One. 2018 May 2;13(5):e0196854. doi: 10.1371/journal.pone.0196854 (PMC5931664; doi:10.1371/journal.pone.0196854)

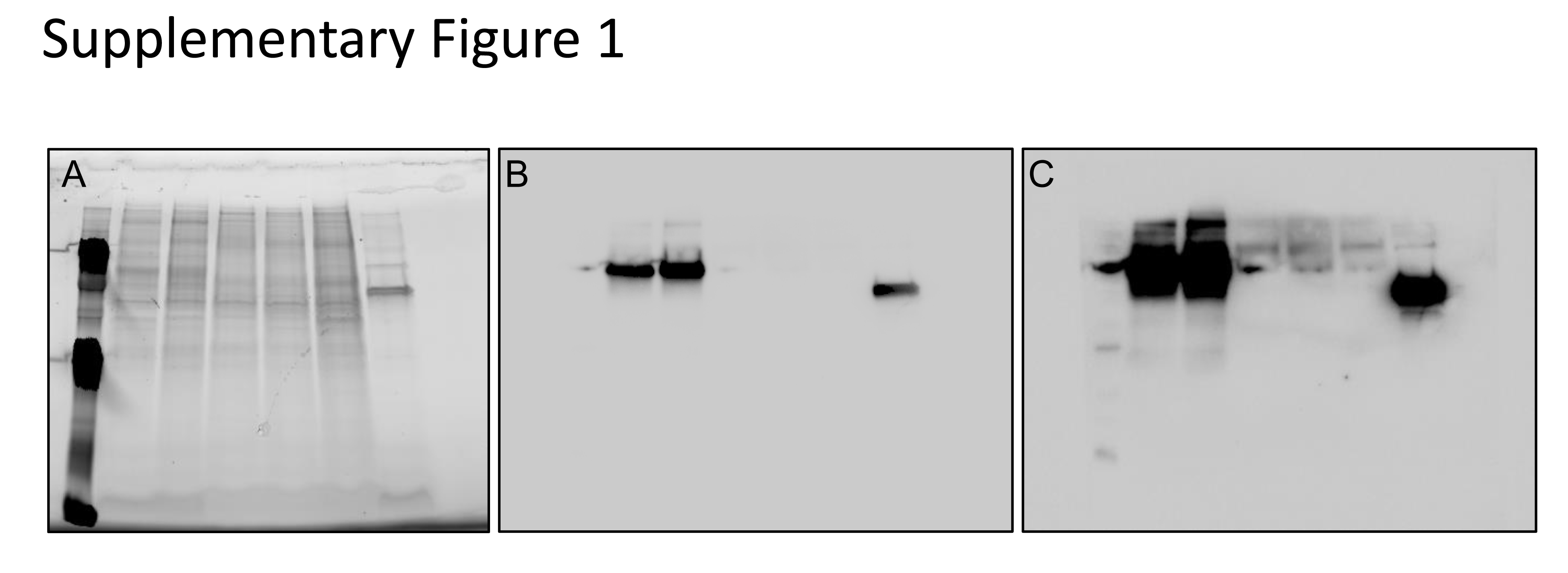

Supplement: S1 Fig — B: Normal exposure time for detection relative to the saturation of the pixel. C: Overexposed SGPL1 immuno blot. (TIF) [file pone.0196854.s001.tif]

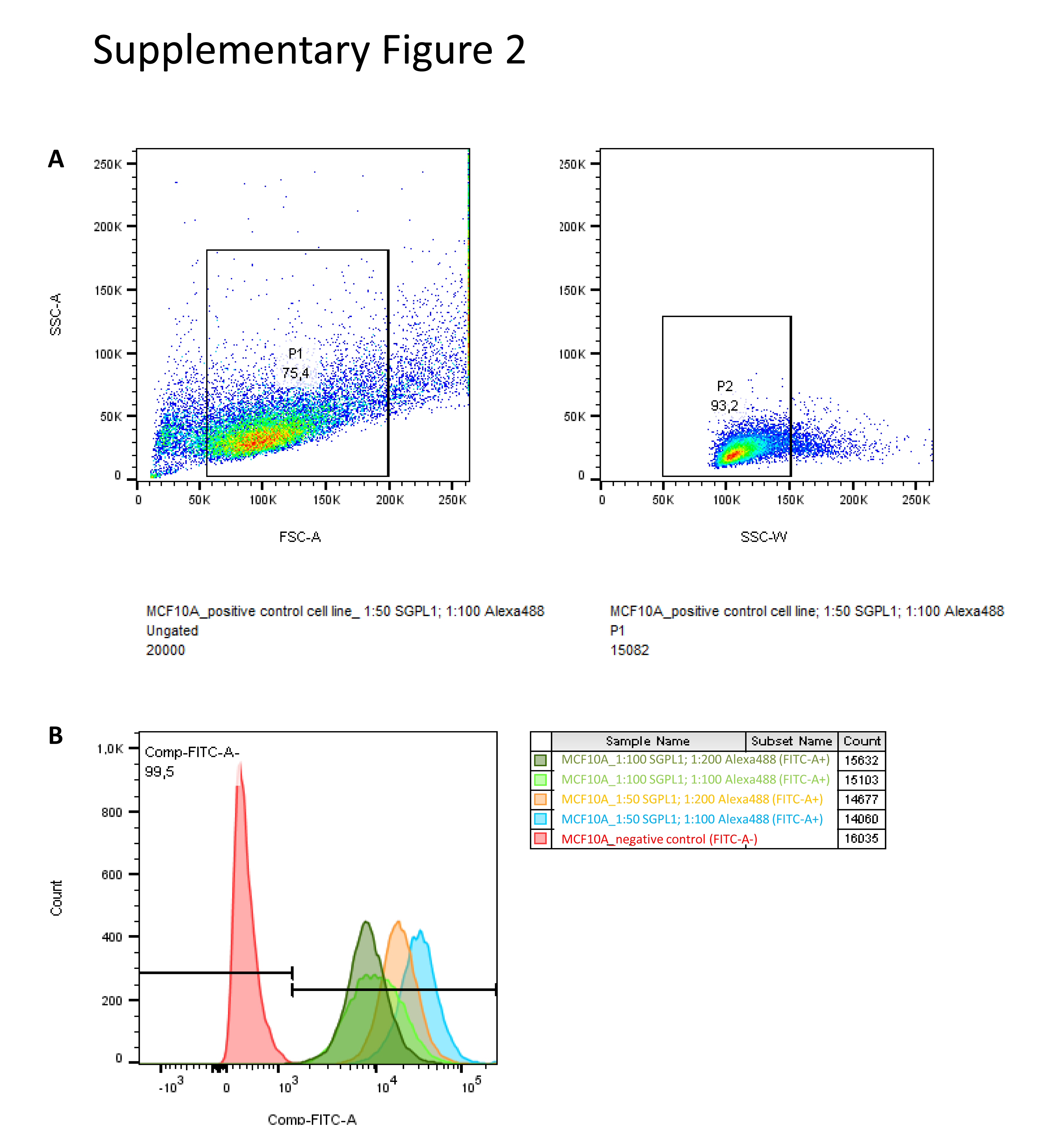

Supplement: S2 Fig — A: Gating of MCF-10A cells. B: Dilution series of primary (anti-SGPL1; 1:50, 1:100) and secondary (anti-rabbit Alexa488; 1:100, 1:200) antibodies to prove specific binding of the SGPL1 antibody. MCF-10A cells incubated with secondary antibody only, functioned as negative control (red histogramm). All signals with an FITC-A+ signal higher than log103 were counted as postive events. (Data for the 1:50 prim./ 1:100 sec. Ab. dilution are shown in the blue histogramm; for 1:50 prim./ 1:200 sec. Ab. dilution in the orange histogramm; for 1:100 prim./1:100 sec. Ab. dilution in the green histogramm and 1:100 prim./ 1:200 sec. Ab. dilution in the dark green histogramm.) The 1:50 dilution of the primary (SGPL1) and 1:100 dilution of the secondary Alexa488-labeled antibody were considered as the effective ones and were used for the experiment. (TIF) [file pone.0196854.s002.tif]

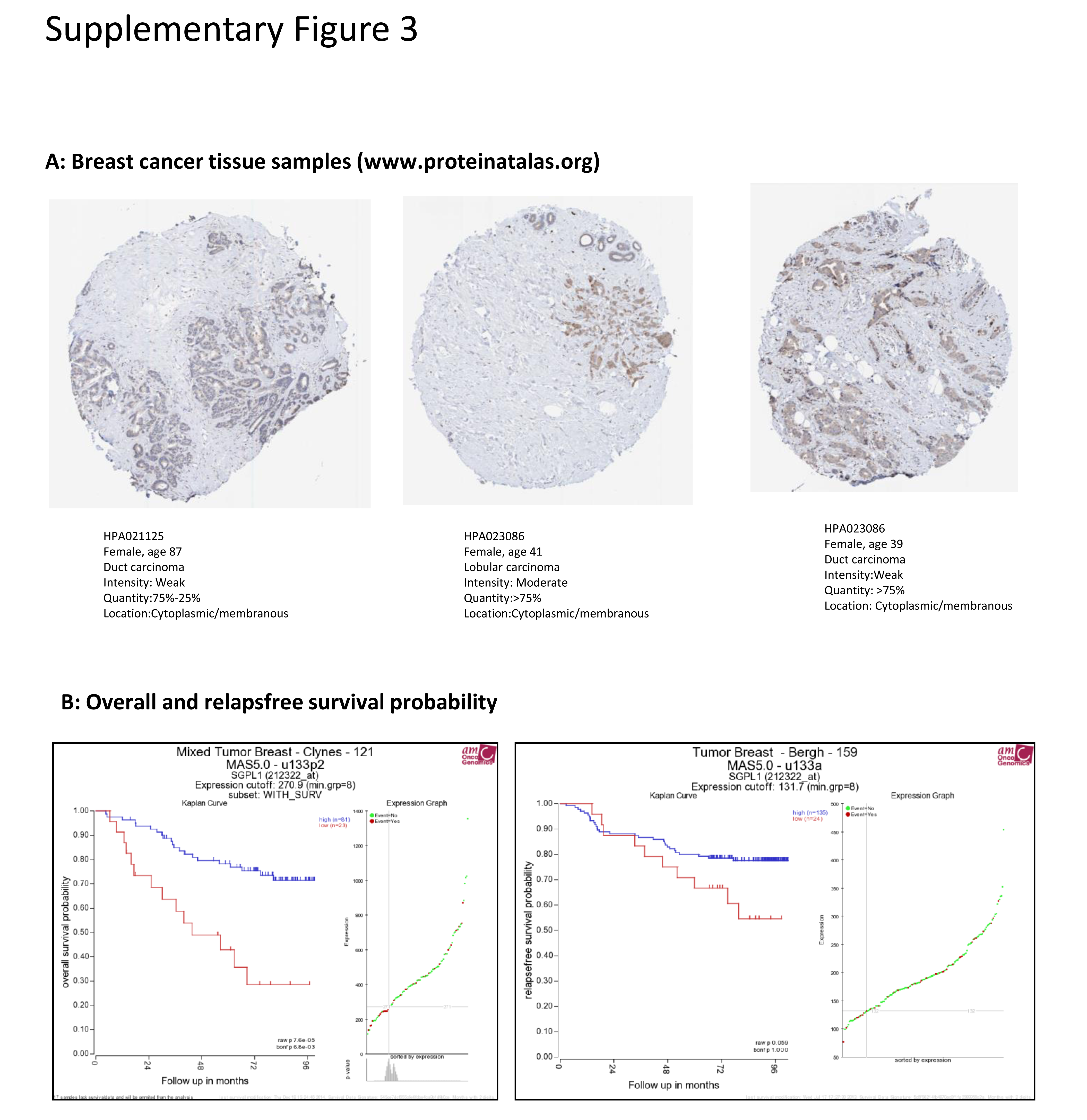

Supplement: S3 Fig — A: SGPL1 expression status in healthy and cancer breast tissues, e.g. http://www.proteinatlas.org/ENSG00000166224-SGPL1/pathology. B: SGPL1 down-regulation is correlated with overall and relapse free survival of breast cancer patients. For example, you can check the online tool R2 for correlation analysis (https://hgserver1.amc.nl/cgi-bin/r2/main.cgi). The following Kaplan Curves demonstrate impressively that low SGPL1 expression leads to poorer overall and relapse-free survival. (TIF) [file pone.0196854.s003.tif]

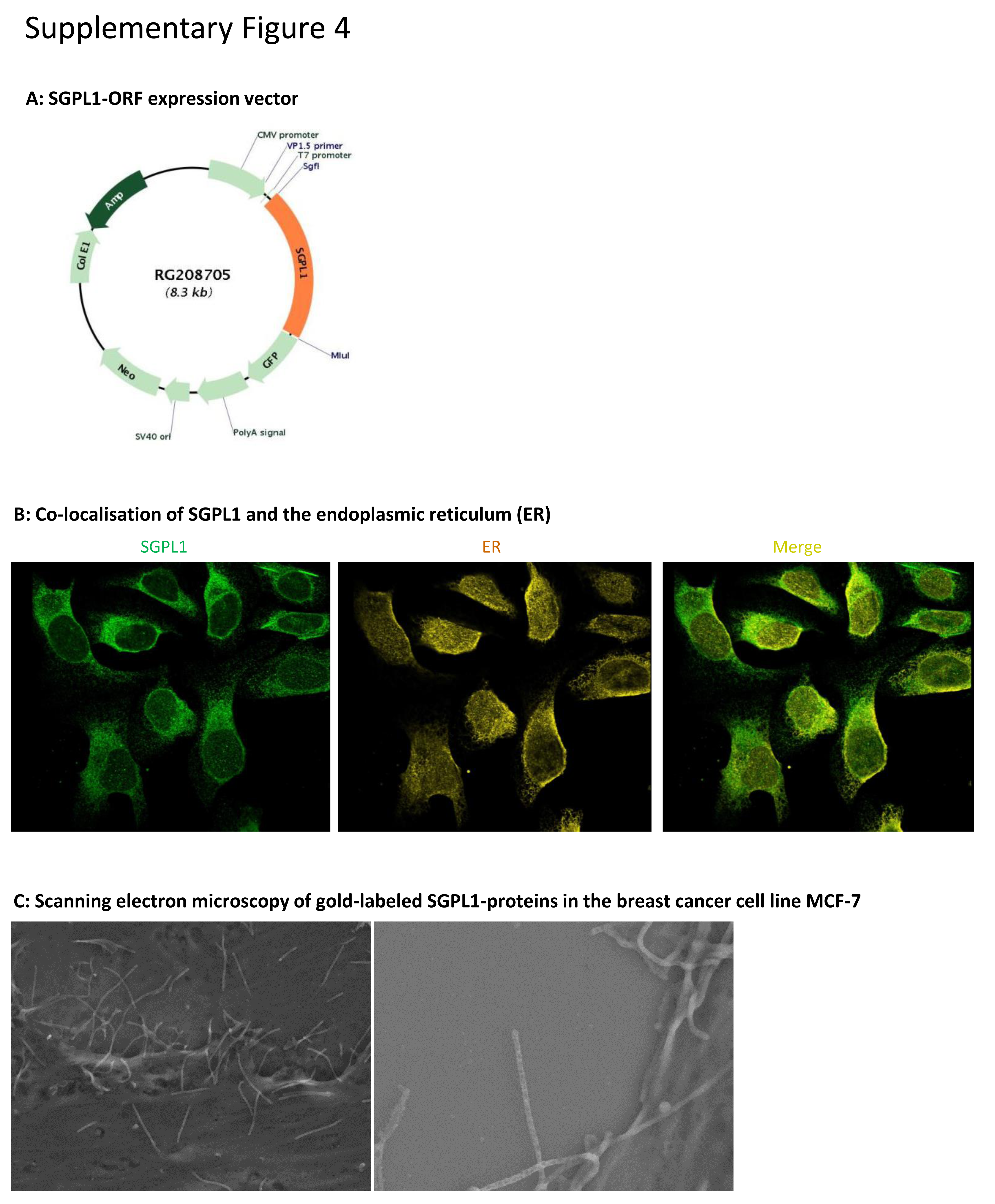

Supplement: S4 Fig — A: Map of the SGPL1-ORF expression vector. B: Co-localization studies of SGPL1 with the endoplasmic reticulum. For further studies see http://www.proteinatlas.org/search/SGPL1. C: Scanning electron microscopy of gold-labeled SGPL1-proteins in the breast cancer cell line MCF-7 showed no signals. (TIF) [file pone.0196854.s004.tif]
